# The SWI/SNF Subunit/Tumor Suppressor BAF47/INI1 Is Essential in Cell Cycle Arrest upon Skeletal Muscle Terminal Differentiation

**DOI:** 10.1371/journal.pone.0108858

**Published:** 2014-10-01

**Authors:** Véronique Joliot, Ouardia Ait-Mohamed, Valentine Battisti, Julien Pontis, Ophélie Philipot, Philippe Robin, Hidenori Ito, Slimane Ait-Si-Ali

**Affiliations:** 1 Université Paris Diderot, Sorbonne Paris Cité, Centre Epigénétique et Destin Cellulaire, UMR7216, Centre National de la Recherche Scientifique CNRS, Université Paris Diderot, Paris, France; 2 Department of Molecular Neurobiology, Institute for Developmental Research, Aichi Human Service Center, Aichi, Japan; National University of Singapore, Singapore

## Abstract

Myogenic terminal differentiation is a well-orchestrated process starting with permanent cell cycle exit followed by muscle-specific genetic program activation. Individual SWI/SNF components have been involved in muscle differentiation. Here, we show that the master myogenic differentiation factor MyoD interacts with more than one SWI/SNF subunit, including the catalytic subunit BRG1, BAF53a and the tumor suppressor BAF47/INI1. Downregulation of each of these SWI/SNF subunits inhibits skeletal muscle terminal differentiation but, interestingly, at different differentiation steps and extents. BAF53a downregulation inhibits myotube formation but not the expression of early muscle-specific genes. BRG1 or BAF47 downregulation disrupt both proliferation and differentiation genetic programs expression. Interestingly, BRG1 and BAF47 are part of the SWI/SNF remodeling complex as well as the N-CoR-1 repressor complex in proliferating myoblasts. However, our data show that, upon myogenic differentiation, BAF47 shifts in favor of N-CoR-1 complex. Finally, BRG1 and BAF47 are well-known tumor suppressors but, strikingly, only BAF47 seems essential in the myoblasts irreversible cell cycle exit. Together, our data unravel differential roles for SWI/SNF subunits in muscle differentiation, with BAF47 playing a dual role both in the permanent cell cycle exit and in the regulation of muscle-specific genes.

## Introduction

The SWI/SNF complex (SWI, mating type switch; SNF, sucrose non-fermenting) is an evolutionarily well-conserved ATPase-powered chromatin-remodeling complex. This complex, composed of a dozen of proteins, is implicated in the sliding or removing of nucleosomes, influencing gene expression regulation, DNA repair and replication (for review [1], [2]). In mammals, SWI/SNF contains one of two related ATPases: Brahma (BRM, encoded by SMARCA2) or Brahma-Related Gene 1 (BRG1, encoded by SMARCA4). In addition, the complex contains BAFs (BRM or BRG1-Associated Factors). The core subunits that are required to reconstitute the SWI/SNF remodeling function in vitro are*:* BAF47/INI1/SNF5/SMARCB1, BAF155/SMARCC1 and BAF170/SMARCC2). Combinatorial assembly of six to eight additional BAFs confers specificity of function to individual SWI/SNF complexes (for review [1], [3]). Some SWI/SNF subunits can participate to other multiprotein complexes also involved in gene transcription regulation, such as N-CoR, WINAC, NUMAC and mSIN3A [2], [4]. SWI/SNF remodeling activity is involved in many physiological processes such as embryonic development, maintenance of pluripotency, cell reprogramming [5], cellular differentiation and pathological processes like tumorigenesis or neurological disorders [6], [7]. Indeed, several SWI/SNF subunits, such as BRG1, BAF250, BAF180 and BAF47, are mutated in various cancer types and some are bona fide tumor suppressors, such as BAF47 [3], [6], [7]. Far from functioning in a similar way in all cell types, SWI/SNF complexes and subunits have a range of specific and context-dependent roles in cell differentiation and proliferation [8]. Indeed, individual SWI/SNF subunits deregulation does not induce the same phenotypes.

BRG1 is required for neuronal differentiation [9], [10], cardiogenesis [11], muscle differentiation (32, 33) and a dominant-negative BRG1 allele blocks myeloid differentiation [12], whereas loss of BRG1 in keratinocytes results in limb patterning defects [13]. BAF47/INI1 plays a crucial role in orchestrating the balance between pluripotency and cell differentiation in embryonic stem cells [14]. Moreover, BAF47/INI1 is a tumor suppressor gene [15], since constitutive mutations have been associated with a strong predisposition to develop malignant rhabdoïd tumors, some of which could be of myogenic origin [16], [17], [18]. One consequence of BAF47/INI1 loss is the activation of gene expression programs that are associated with proliferation [19], [20], [21], [22]. However, other studies report that BAF47/INI1 is also implicated in differentiation of hepatocytes [23] and adipocytes [24]. Among other BAF subunits, BAF53/ARP4/ACTL6 is a nuclear Actin Related Protein play different roles [25]. Indeed, its interaction with the core SWI/SNF complex is required for the maximal ATPase activity of BRG1 and for the association of the SWI/SNF complex with chromatin [26], [27]. Two distinct BAF53 genes have been described, BAF53a and BAF53b. This subunit is an essential regulator of adult stem cell function [25], [28]. BAF53 is involved in self renewal of progenitor and stem cells, like long-term HSC (hematopoietic stem cells), myeloid progenitor cell [29], neural stem and progenitor cells [10] and epidermal progenitor cells [30].

Several studies have shown the involvement of SWI/SNF complex(es) in skeletal muscle terminal differentiation. There are functional interactions between different master myogenic bHLH (basic Helix-Loop-Helix family) transcription factors (including MyoD, Myf5, MRF4 and myogenin) and many SWI/SNF subunits during myoblast-to-myotube transition [31], [32]. Indeed, dominant-negative forms of BRM or BRG1 inhibited muscle marker expression and terminal differentiation [33]. In contrast, BAF57 has been shown to act as a partner of zinc finger-containing factor Teashirt (TSHZ3) to repress MyoD-dependent myogenin expression and regulate skeletal muscle differentiation [34]. In addition, BAF60c and MyoD interact on the regulatory elements of MyoD-target genes in myoblasts [35]. Phosphorylation of BAF60c by p38α kinase induces SWI/SNF complex recruitment to muscle gene promoters through interaction with the BAF60c/MyoD complex and allows transcriptional activation of muscle target genes [35]. Lack of BAF60c prevents myogenic programming [36], [37], [38].

Although SWI/SNF complexes have been implicated in skeletal muscle terminal differentiation, the role of BAF47 in this process remained elusive. The aim of the present study is to unravel, in an *ex vivo* model of skeletal muscle terminal differentiation, specific roles of BAF47 compared to BRG1 and BAF53a, which roles have not been studied. Our data show that knockdown of one of these three subunits induced impairment in ex vivo skeletal muscle terminal differentiation. Most importantly, this impairment seems to involve more than their roles within the SWI/SNF complexes. Our results unravel crucial and specific roles for these subunits in the balance between proliferation and terminal differentiation in skeletal muscle.

## Materials and Methods

### MyoD complex purification and characterization

Flag-HA-MyoD complex purification from HeLa-MyoD cells was performed as described in [39]. Briefly, we used retroviral transduction strategy to establish HeLa-S3 cell line expressing double-tagged Flag-HA-MyoD and a control cell line transduced with the empty pREV vector. We carried out double-affinity purification of Flag-HA-MyoD from HeLa cells using either nuclear soluble or chromatin fractions. Double-immunopurified complexes were resolved on SDS-PAGE bis-Tris acrylamide. In the latest, bands corresponding to proteins were cut from the gel, trypsin-digested and identified by mass spectrometry (MS) (41).

### siRNA and Transfection

SiRNAs were purchased either from Dharmacon or Sigma Aldrich. C2C12 cells were transfected with 100 nM final siRNA concentration using HighPerfect (Qiagen) according to the manufacturer’s protocol. SiRNA sequences used were:

Brg1-2: CAAACUGGGCGUAUGAAUU; Brg1-4: GAGACUAUCCUCAUUAUUC; BAF53a-1: CCACCAAGUAUGCGGUUGA; BAF53a-3: GCUUUAGUACACUCAGGAA. BAF47-2: GUGGGAAACUACCUGCGUA; BAF47-4: GAUAGGAACACAAGGCGAA. Control: ACUUAACCGGCAUACCGGCTT.

### RT-PCR

Reverse transcription was performed with “high capacity cDNA reverse transcription” kit (Life Technologies). qPCRs were performed with SYBR® Green master mix (Life technologies) on the 7500HT machine (Applied Biosystems). Cyclin D1 specific primers were: Fw: 5'TTCTTTCCAGAGTCATCAAGTGTGA and Rev: 5' CCAGAAGGGCTTCAATCTGTTC). Cyclophilin A specific primers were: Fw: GTCAACCCCACCGTGTTCTT, Rev: CTGCTGTCTTTGGGACCTTGT.

### Cell culture

C2C12 cells were cultured under standard conditions, as previously described [40]. Growth media (GM) = DMEM with 15% of fetal calf serum (FCS). Differentiation media (DM) = DMEM with 2% horse serum. DM was added 24 h after siRNA transfection.

### Immunofluorescence and BrdU staining

C2C12 cells were plated in chamber slides (Nunc Lab-Tek II Chamber Slide System, Thermo Scientific) and transfected with siRNA. After 72 h of incubation in DM, cells were washed twice with PBS and fixed with 4% paraformaldehyde for 20 mn at room temperature. Fixed cells were permeabilized with Triton X-100 0.2% for 30 mn and blocked with blocking buffer (1% normal goat serum, BSA 1% in PBS) for 1 h and incubated 1 h with rabbit-anti-MCK. After 3 washings with PBS, cells were incubated for 30 min with secondary red anti-rabbit antibody (1∶500) (Alexa Fluor 488 Goat anti-Rabbit).

Cell proliferation was assessed using a single BrdU/nuclear-labeling assay. C2C12 cells were plated in chamber slides; BrdU (5-Bromo-2'-DeoxyUridine) incorporation was monitored in two culture conditions after 96 h in DM and 96 h of DM followed by 12 h in GM. BrdU was added in cell culture media at a final concentration of 10 µM during 2 h. The immunostaining was performed according to the manufacturers’ instructions (Roche Applied Science). Finally, cells were stained with DAPI (4′,6-diamidinole-2-phenolindole) (Sigma Aldrich) and analyzed by fluorescence microscopy (Leica DMI 6000 B). BrdU and DAPI staining were counted over 2000 cells per condition. BrdU index was calculated as the ratio of BrdU positive nuclei to DAPI-labeled nuclei.

### Preparation total cellular extracts

Cells were scraped in a minimal volume of PBS and centrifuged 2 min at 400 g. Cell pellets were suspended in RIPA (50 mM Tris-HCl pH 7.5; 150 mM NaCl; 1% NP-40, 0.5% Na-Deoxycholate, 0.1% SDS, 1 mM EDTA). After 10 mn on ice, the lysates were sonicated on “High”, for 7.5 min of cycles 15 sec –1 min OFF with the BioRuptor (Diagenode) then centrifuged 10 min at 13000 rpm to harvest the total cellular extracts (supernatants). Protein concentration was estimated with the BCA kit (Pierce, Thermo Scientific).

### Glycerol gradient

300 to 400 µg of nuclear extracts were deposited in a 4.5 ml of 11% - 33% glycerol gradient and centrifuged in a MLS50 rotor (Optima MAX-XP, Beckman Coulter) at 49 000 rpm for 14 h. 400 µl fractions were collected, precipitated in TCA and washed in acetone. Proteins were analyzed by western blot.

### Endogenous co-immunoprecipitation (co-IP)

C2C12 cell pellets in GM and after 24 h of DM were lysed in hypotonic buffer (20 mM HEPES pH 7, 0.15 mM EDTA, 0.15 mM EGTA, 10 mM KCl) with freshly added spermine, spermidine (0.15 mM, 0.5 mM, respectively) and protease inhibitor cocktail (Roche Applied Science). Cell lysates were centrifuged at 9000 rpm for 7 mn. The pellets were suspended in sucrose buffer (20 mM Tris pH 7.65; 60 mM NaCl; 15 mM KCl; 0.34 M Sucrose) and then in high salt buffer (20 mM Tris-HCl pH 7.65; 0.2 mM EDTA; 25% glycerol; 900 mM NaCl; 1.5 mM MgCl_2_) to a final NaCl concentration of 300 mM. The nuclear extracts were treated with Micrococcal nuclease (0.0025 U/µl) at 37°C during 10 mn and sonicated during 10 mn at high frequency. The lysates were ultracentrifuged at 40000 rpm for 30 mn (Optima MAX-XP, Beckman Coulter) and pre-cleared during 1 h. Immunoprecipitations were carried out overnight at 4°C using 5 µg of each antibody. The second day, the immunocomplexes are washed four times in wash buffer (50 mM Tris-HCl, pH 7.65, 150 mM NaCl, Triton X-100 0.5%) and the proteins were eluted in NuPAGE LDS Sample Buffer (4X) (Life Technologies) at 96°C during 5 min. Finally, the immunoprecipitates were examined by western blot.

### Chromatin Immunoprecipitation (ChIP)

We performed a two steps crosslinking by adding DSG (Di-Succinimidyl Glutarate; Santa Cruz) at a final concentration of 2 mM for 45 min at room temperature then Formaldehyde (Sigma) to a final concentration of 1% for 10 min at room temperature. Crosslinking was stopped by addition of glycine at a final concentration of 0.125 M. Fixed cells were washed and harvested with PBS. Chromatin was prepared by two subsequent extraction steps (10 min, 4°C) with: Buffer 1 (50 mM HEPES/KOH pH 7.5; 140 mM NaCl; 1 mM EDTA; 10% Glycerol; 0.5% NP-40; 0.25% Triton) and Buffer 2 (200 mM NaCl; 1 mM EDTA; 0.5 mM EGTA; 10 mM Tris pH 8). Nuclei were then pelleted by centrifugation, resuspended in Buffer 3 (50 mM Tris pH 8; 0.1% SDS; 1% NP-40; 0.1% Na-Deoxycholate; 10 mM EDTA; 150 mM NaCl) and subjected to sonication with Bioruptor Power-up (Diagenode) yealding genomic DNA fragments with a bulk size of 150–300 bp. Chromatin was precleared with Protein A/G ultralink beads (53133, Pierce) for 2 h at 4°C and immunoprecipitation with the specific antibodies carried out overnight at 4°C. Immune complexes were recovered by adding pre-blocked protein A/G ultralink beads and incubated for 2 h at room temperature. Beads were washed twice with Low salt buffer (0.1% SDS; 1% Triton; 2 mM EDTA; 20 mM Tris pH 8; 150 mM NaCl), twice with High salt buffer (0.1% SDS; 1% Triton; 2 mM EDTA; 20 mM Tris pH 8; 500 mM NaCl), once with LiCl wash buffer (10 mM Tris pH 8.0; 1% Na-deoxycholate; 1% NP- 40, 250 mM LiCl; 1 mM EDTA) and twice with TE +50 mM NaCl. Beads were eluted in TE +1% SDS at 65°C and cross-link was reversed O/N at 65°C. The eluted material was phenol/chloroform-extracted and ethanol-precipitated. DNA was resuspended in water. Cyclin D1 primers are located over the TSS (Fw: CTTTTCTCTGCCCGGCTTT; Rev: ACTCCCCTGTAGTCCGTGTGA).

### Western blotting

For western blotting, protein samples were resolved on pre-cast NuPage 4–12% bis-Tris acrylamide gradient SDS-PAGE gel (Life Technologies). Proteins were then transferred onto nitrocellulose membrane during 2 h at 800 mA in phosphate transfer buffer. Membranes were blocked 1 h in PBS-0.2% Tween 20, 10% skimmed milk and incubated overnight at 4°C with primary antibodies. Membranes were incubated with the appropriate secondary antibodies coupled to HRP (horse radish phosphatase ) and revealed using West Dura from Pierce (Thermo Scientific) and ChemiSmart 5000 system (Vilber Lourmat) and quantified using Chemicapt software.

### Antibodies

The anti-MyoD (M-318 sc760, C-20 sc304, 5.8A sc32758), anti-myogenin (M-225, sc-576), anti-cyclin A2 (C-19, sc-596), anti-cyclin D1 (72–13G, sc-450), anti-cyclin D3 (C-16, sc-182), were purchased from Santa Cruz Biotech. Anti-BRG1 (clone 3G4) is from Active Motif, anti-BAF47 (612111) and anti HDAC3 (histone deacetylase) (611125) from BD Biosciences, anti-PRMT5 (07-405) from Merck Millipore and anti-KAP1 (ab10483) is from Abcam. Anti-BAF53a (A301-391A) and anti-BAF170 (A301-038A) are from Bethyl. Anti-MHC (myosin heavy chain) and anti-α-tubulin (α-tub) antibodies were purchased from Sigma Aldrich. Anti-MCK (muscle creatine kinase) is developed by Dr. H Ito. For ChIP experiments, we used anti-BRG1 (ab110641) and anti-BAF47 (ab12167) from Abcam.

## Results

### MyoD interacts with the SWI/SNF complex

**Figure 1:**
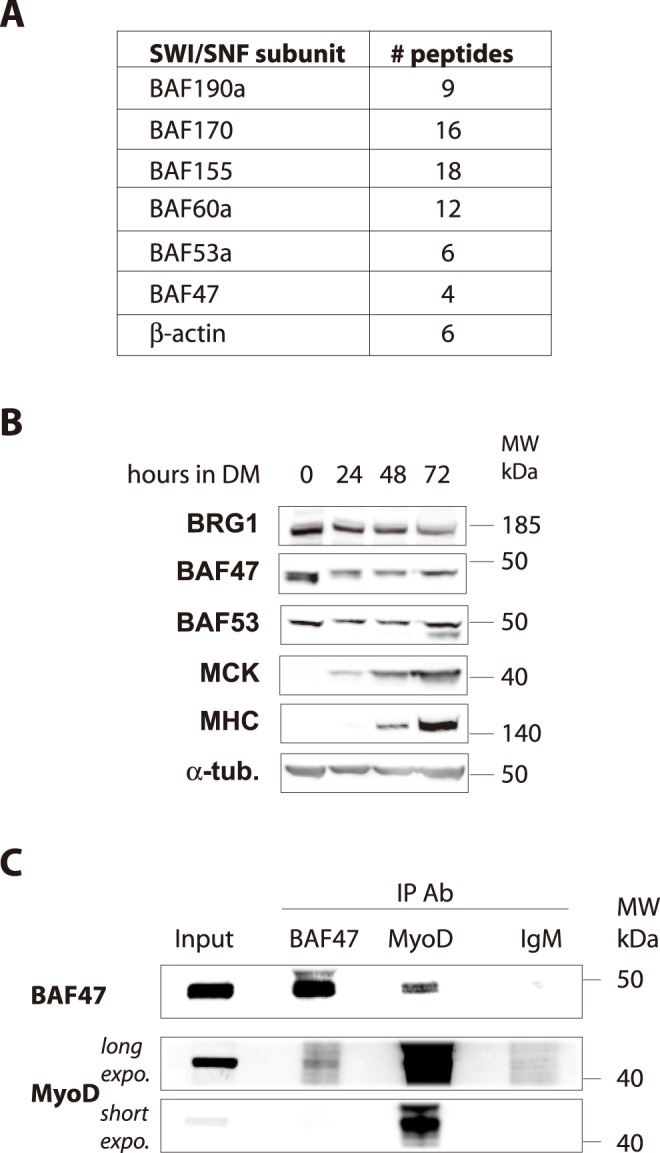
SWI/SNF complex subunits interact with MyoD. **A.** SWI/SNF subunits identified by mass spectrometry in TAP-tag purified MyoD complex. **B.** Soluble nuclear extracts from proliferating C2C12 myoblasts were immunoprecipitated with BAF47 or MyoD antibodies, or control IgM. The resulting precipitates were analyzed by western blot (WB) with the indicated antibodies.. α-tub: alpha-tubulin, loading control; IP: immunoprecipitation; Ab: antibody. For some WB lower (short expo.) and higher (long exp.) exposure times are shown. **C.** C2C12 myoblasts were cultured in GM (0) then cells were placed in DM and harvested after 24 h, 48 h or 72 h. Total protein extracts were analyzed by WB with the indicated antibodies.

In an attempt to characterize MyoD protein partners, we have carried out double-affinity purification of HA-Flag-tagged MyoD stably expressed in HeLa cells as described [39], [41]. Mass spectrometry (MS) analysis revealed some already known partners of MyoD, such as PC4, Pbx1 and E12/E47 (data not shown) and new partners, such as HP1 and CBFβ that have been studied previously [39], [41]. Among the already known partners of MyoD, we confirmed the presence of different core subunits of the SWI/SNF chromatin-remodeling complex. Namely, BAF190a, BAF170, BAF155, BAF60a, BAF53a, BAF47 and β-actin (Figure 1A). HeLa cells are resistant to MyoD-induced transdifferentiation since they do not express BAF60c, which is required for MyoD-mediated activation of gene expression in muscle cells [35]. From our results it appears that BAF60a isoform is, anyhow, part of the MyoD complex in HeLa cells suggesting that, if it cannot support MyoD-induced transcription, it is for another reason than its participation to the MyoD/SWI/SNF complex activities.

Skeletal muscle terminal differentiation is a two-step process, associating an irreversible cell cycle exit followed by the induction of muscle-specific genes [42], [43], [44], [45]. There are many studies describing BRG1, BRM, BAF60c and BAF53a involvement in the two steps of skeletal muscle terminal differentiation [33], [46], whereas the BAF47 core subunit, a well-known tumor suppressor, has not been studied individually in this process. BRG1, BAF47 and BAF53a are expressed in proliferating C2C12 myoblasts. BAF53a expression levels are stable during differentiation while BRG1 and BAF47 levels decrease during muscle terminal differentiation (Figure 1B).

We sought to more precisely investigate if BAF47 could play a specific role that would regulate the permanent cell cycle exit. Thus, we first confirmed that BAF47 and MyoD can interact by co-immunoprecipitation (co-IP) experiments from nuclear soluble extracts of proliferating C2C12 murine myoblasts. Immunoprecipitation of MyoD co-precipitated BAF47 (Figure 1C). Reciprocally, immunoprecipitation of BAF47 co-precipitated MyoD but less efficiently (Figure 1C).

Together, these data show that MyoD interacts with the tumour suppressor subunit BAF47 in myoblasts.

### SWI/SNF chromatin remodeling complex subunits are differently involved in muscle differentiation

Throughout the study, we focused on BAF47 tumor suppressor subunit and performed comparative studies with BAF53a and the catalytic subunit BRG1, since the latter has been well studied in muscle terminal differentiation (33). We analyzed the effect of RNAi-mediated downregulation of these subunits in C2C12 mouse myoblast cell line. We first used 4 different siRNAs for each subunit (Figure S1) and carried on the study by the use of the best ones (one or two). We routinely induced around 50% of downregulation for the different SWI/SNF subunits. We have performed growth curves in high serum conditions and did not notice any significant effects of the different siRNAs on C2C12 myoblast proliferation (data not shown).

**Figure 2:**
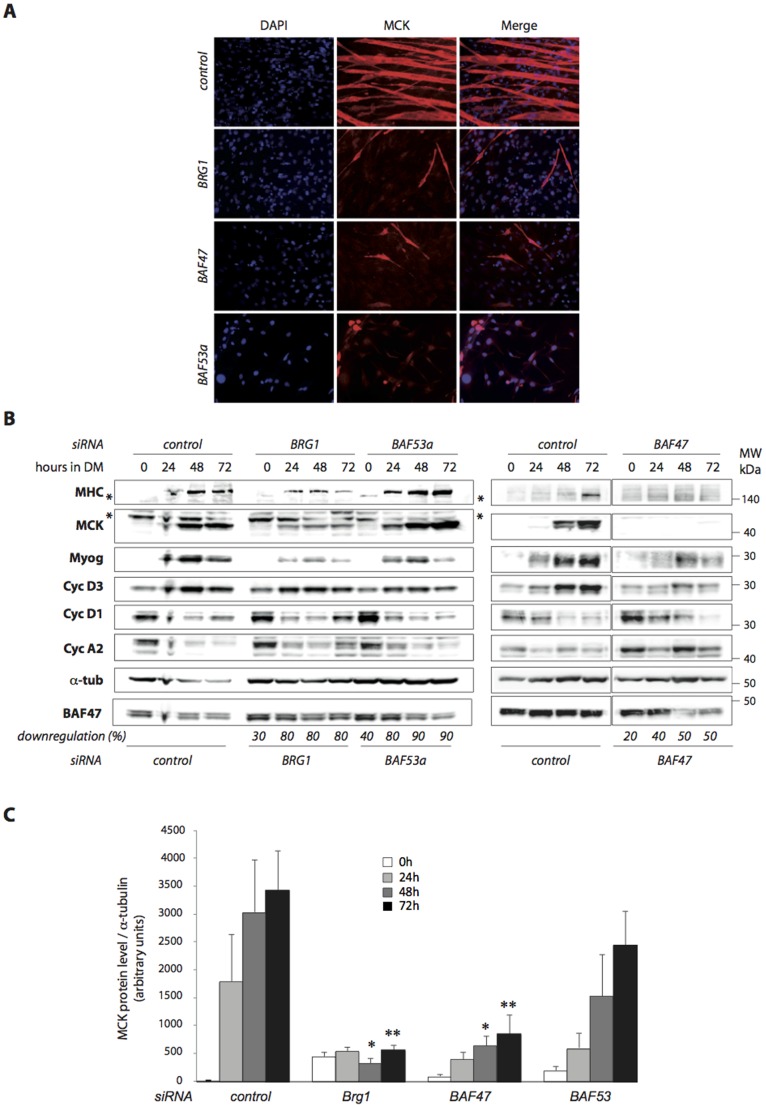
SWI/SNF complex subunits are differently involved in skeletal muscle terminal differentiation. **A.** C2C12 myoblasts were transfected with control, BRG1, BAF53a or BAF47 siRNAs. Immunofluorescence analyses using anti-MCK antibody were performed after 72h in DM. Cells were DAPI-stained prior to fluorescent microscopy analyses (magnification x20). **B and C.** C2C12 myoblasts were transfected with control, BRG1, BAF53a or BAF47 siRNAs. 24 h post-transfection (0) cells were placed in DM and harvested after 24 h, 48 h or 72 h. **B.** Total protein extracts were analyzed by WB with the indicated antibodies. *: non-specific bands. For each time point, % of protein siRNA-induced downregulation compared to the same time from control scrambled siRNA is indicated at the bottom of the western blot (see Figure S2 for additional WB analyses). **C.** Quantification of MCK levels from 3 to 8 independent western blot. Error bars represent standard error of the mean (SEM). For each time point, statistics were calculated compared to the same time point from control. Significative p-values (Student T-test, two tailed, unpaired) are indicated * = <0.05; ** = <0.01.

Downregulation of BRG1, BAF47 or BAF53a impaired terminal muscle differentiation (Figure 2). Note that downregulation of one SWI/SNF subunit does not affect the stability/expression of the other studied subunits (Figure S2 and data not shown). In BRG1-, BAF47- or BAF53a-downregulated myoblasts, no multinucleated myotubes were observed in differentiation conditions compared to control myoblasts (Figure 2A). We realized immunofluorescent staining to detect expression of MCK, a muscle specific marker. For BRG1- or BAF47-downregulated myoblasts, no expression of MCK was detected except for few positive elongated cells, which correspond to untransfected myoblasts (Figure 2A). While in BAF53a-downregulated myoblasts, the majority of the cells exhibited a positive staining for MCK, even if the signal is weaker compared to the control myotubes (Figure 2A). For those siRNA-transfected myoblasts that cannot differentiate upon serum removal we frequently observed an increase in cell death, especially in BAF53 KD, that could lead to a decrease in cell number after 72 h in DM. To further confirm and analyze the misregulation of muscle specific genes, we performed western blot analyses (Figure 2B). We studied myogenin, MCK and MHC emergence, which are respectively early, mid or late skeletal muscle differentiation markers. In an attempt to characterize the consequences of siRNA-mediated downregulations on cell cycle, we also studied three cyclins whose expression is either negatively regulated upon cell cycle exit (cyclin D1 and cyclin A2) or positively regulated (cyclin D3). Using this approach, we confirmed that myoblasts depleted for BRG1 or BAF47 and induced to differentiate exhibit very low levels of the three muscle markers compared to control myoblasts (Figure 2B). For BAF53a-depleted myoblasts, myogenin, MCK and MHC are almost normally expressed (Figure 2B). Since there is some variability between experiments due to different downregulation efficiencies, we performed statistical analyses of 3 to 8 independent experiments confirmed that the defect in the induction of MCK expression upon muscle differentiation when BRG1 or BAF47 were downregulated is significant (Figure 2C), while the lowest levels of MCK in BAF53-downregulated cells is not significant.

We also analyzed cyclin expression profiles. Some differences were noticed upon BAF47 downregulation (Figure 2B) but they were not statistically significant (data not shown). Altogether, these data suggest subtle differences in the roles of individual SWI/SNF subunits during skeletal muscle terminal differentiation. Indeed, downregulation of BAF53a affects drastically the cellular phenotype of differentiation without significantly affecting regulation of cyclins and muscle markers, while downregulation of BAF47 or BRG1 affects differentiation and inhibits expression of muscle-specific genes.

### BAF47, but not BRG1 nor BAF53a, negatively regulates cell cycle exit

**Figure 3:**
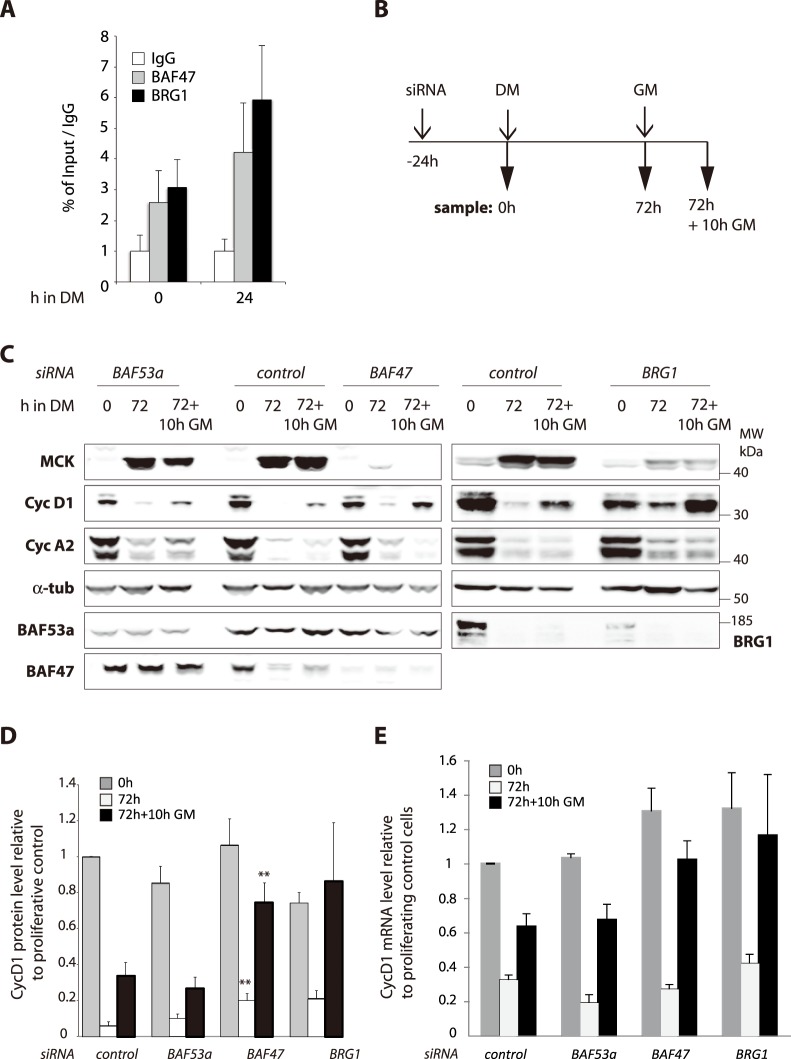
Downregulation of BAF47 alters muscle terminal differentiation and cell cycle exit. **A.** BAF47 and BRG1 occupancy at *cyclin D1* promoter. ChIP-qPCR analyses of BAF47 and BRG1 in myoblast in proliferating C2C12 cells and at 24 h of differentiation. The immunoprecipitated material was quantified by qPCR, and results are expressed as fold enrichment of the % of Input of BAF47 or BRG1 ChIP over % of Input of the IgG average. Data are represented as mean ±SEM, n = 3. **B.** Scheme of the timing for samples collection. C2C12 myoblasts were transfected with control, BRG1, BAF53a or BAF47 siRNAs and samples prepared either 24 h after transfection (0) or after 72 h in DM (72) or 10 h after the switch back to GM (72+10 h GM). **C.** Total protein extracts were analyzed by WB with the indicated antibodies. **D.** Quantification of cyD1 levels from 3 to 8 independent WB. Data are expressed compared to control 0 h. Error bars represent SEM. For each time point, statistics were calculated compare to the same time point from control. Only significative p-values (Student T-test, two tailed, unpaired) are indicated ** = <0.01. **E**. Total RNA was isolated, reverse transcribed using random primers and used as templates for PCR amplification with cyclin D1-specific primers and normalized to cyclophilin A specific primers. Data are expressed compared to control 0 h.

BAF47-downregulated myoblasts seem to have a delay in cell cycle exit, even if no significant misregulation of cyclins was observed during the first days of differentiation (Figure 2B). In some cases, it has been shown that SWI/SNF complex could be involved in Cyclin D1 regulation. We first analyzed the presence of some SWI/SNF subunits on *Cyclin D1* promoter by ChIP-qPCR. Our data indicate that BRG1 and BAF47 could be detected on *Cyclin D1* promoter where they slightly increase after differentiation induction (Figure 3A). This delayed cell cycle exit correlated with the absence of expression of muscle markers. Thus, to better define effects of SWI/SNF subunits downregulation on the cell cycle exit and terminal differentiation, C2C12 myoblasts were transfected with siRNAs, switched to differentiation medium (DM) for 3–4 days and then switched back to growth media (GM), as described in Figure 3B. Control differentiating myoblasts expressed almost no cyclin D1 after few days in DM, and the residual level of cyclin D1 detected after serum addition is likely to come from cells that are refractory to differentiation (Figure 3C). We observed very similar results in BAF53a-depleted myoblasts (Figure 3C). In the absence of serum, differentiating myoblasts treated with BRG1 or BAF47 siRNAs expressed slightly more cyclin D1 than the control cells. However, treatment of these cells with serum induced a much higher level of cyclin D1 than in control cells (Figure 3C). For cyclin A2 expression pattern, we did not observe any differences between control cells and downregulated ones (Figure 3C). We quantified on western blot the cyclinD1 levels for 3 to 10 independent experiments (Figure 3D). The differences observed for BAF47 downregulation were statistically significant but this is not the case for BRG1 downregulation (Figure 3D).

RT-qPCR analyses allowed us to show that the increase in cyclin D1 protein is correlated with an increase of mRNA (Figure 3E). These results suggest that in cells depleted for BRG1 or BAF47, the *cyclin D1* promoter is not efficiently silenced upon differentiation. Conversely, BAF53a seems not essential for the SWI/SNF role in transcription regulation since cyclins or muscle marker genes behave similarly in BAF53a-depleted and control cells.

**Figure 4:**
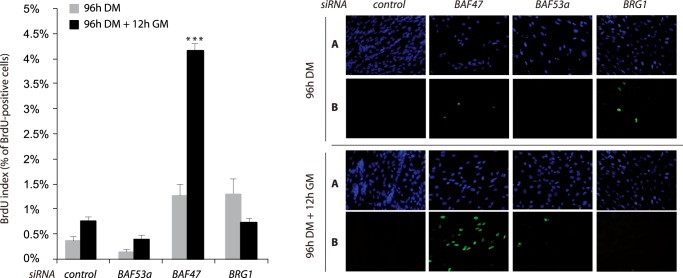
Effect of SWI/SNF subunits BAF47, BAF53a and BRG1 knockdown on the irreversible cell cycle exit. C2C12 myoblasts were transfected with control, BRG1, BAF53a or BAF47 siRNAs and cells were labeled with BrdU either after 96 h in DM or 12 h after the switch back to GM (96+12 h GM). We show one representative of 3 independent experiments. Left panel: BrdU indexes ratio of BrdU positive nuclei/DAPI labeled nuclei) are shown in the histogram, error bars represent SEM from 3 quantifications of the presented experiment (*p<10^−5^ according to Student test (two tailed, unpaired)). Right panel: A: DAPI staining (blue) (x20) and B: BrdU immunostaining (green) (x20).

To analyze cell cycle exit in these specific conditions, we performed BrdU staining experiments in BRG1-, BAF47- or BAF53a-depleted C2C12 cells, after 96 h in DM and 96 h DM +12 h GM. SiRNA efficiency was analyzed by western blot (Figure S2). We define the BrdU index as the ratio between the number of BrdU-positive nuclei/number of DAPI-labeled nuclei. In proliferating control myoblasts the BrdU index is usually around 6%. As for the experiment presented in Figure 2, we always induced differentiation at the same myoblast density but for siRNA-transfected myoblasts that cannot differentiate upon serum removal we frequently observed an increase in cell death, especially in BAF53 KD, that could lead to a decrease in cell number after 96 h in DM. After 96 h in DM, BRG1- and BAF47-downregulated cells keep a minor proliferating rate with BrdU indexes of 1,3% and 1,26%, respectively (Figure 4). For this time point, control cells and BAF53a-downregulated cells have almost null BrdU indexes, of 0,35% and 0,13% respectively. After 96 h in DM, we changed to high serum containing media during 12 h. We observed an important increase of BrdU index up to 4,15% only for BAF47-downregulated cells, while the BrdU index for BRG1-downregulated cells keep decreasing to 0.72%. At 96 h in differentiation medium, BrdU indexes show some residual cycling cells that could be cells resistant to differentiation. But at 96 h DM +12 h GM, we observe a significant increase in BrdU index only for BAF47 KD cells. This important increase in cycling cells cannot be explained by cells resistant to differentiation since we did not observe this for BAF53 or BRG1 KD cells, while they are also undifferentiated. The decrease of BrdU index for BRG1-downregulated cells could reflect a delayed but definitive cell cycle exit (Figure 4). These results clearly demonstrate that downregulation of BAF47, but not BRG1 or BAF53a, prevents the irreversible cell cycle withdrawal.

Altogether, these data suggest that SWI/SNF subunits could play different roles during the proliferation/differentiation switch, with BAF47 being essential in the permanent cell cycle exit.

### BAF47 and BRG1 interact with transcriptional co-repressors

Our data show that downregulation of BRG1 or BAF47 have different outcomes since both inhibit muscle marker genes expression but only BAF47 downregulation perturbs irreversible cell cycle exit. We have also shown that levels of either BAF47 or BRG1 decrease upon myoblasts to myotubes differentiation. In addition to their involvement in the SWI/SNF complexes, BRG1 and BAF47 can also participate in different of regulatory complexes, such as WINAC, NUMAC, mSin3A/HDAC and N-CoR (nuclear receptor co-repressor) [4].

**Figure 5:**
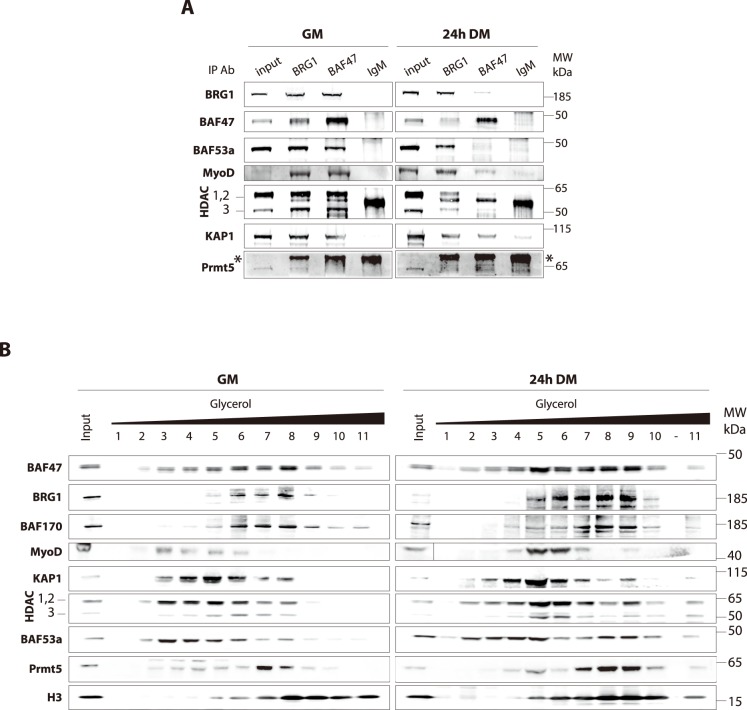
BRG1 and BAF47 interact with SWI/SNF and N-CoR-1 complex components in a differentiation-dependent manner. **A.** Nuclear extracts from proliferating (GM) or differentiating C2C12 myoblasts (24 h DM) were used for immunoprecipitation (IP) with antibodies against BRG1 or BAF47, or with normal rabbit IgG as a negative control. The resulting precipitates were analyzed by WB with the indicated antibodies. Nuclear extracts (Inputs, 1% of input extracts) were loaded to assess endogenous protein levels. *: non-specific IgG band. **B.** Nuclear extracts from proliferating (GM) or differentiating C2C12 myoblasts (24 h DM) were fractionated on glycerol gradient ranging from 11% (fraction 1) to 33% (fraction 11); (-) empty lane. Fractions were collected and analyzed by WB using the indicated antibodies. Nuclear extracts (Inputs, 2.5% of input extracts) were loaded to assess endogenous protein levels.

To investigate for transcriptional co-regulators that could interact with BRG1 and BAF47 in our *ex vivo* model of skeletal muscle terminal differentiation, we performed co-IP assays. We analyzed complex composition in proliferating *versus* early differentiating myoblasts to avoid the bias of decrease in BRG1 and BAF47 during late differentiation. Our results confirmed the association of BRG1, BAF47 and BAF53a in proliferating myoblasts. We also confirmed the association of BRG1 and BAF47 with MyoD, which is clearly detected in IP even if it is weakly expressed at this stage (Figure 5A). We have also easily detected co-precipitation of HDACs and KAP1, involved in the N-CoR-1 complex, to a similar extent with both BRG1 and BAF47 (Figure 5A). We also tested PRMT5 interaction with BRG1 and BAF47 since it is known that these proteins are co-involved in mSin3A repressive complexes [47]. According to Figure 5A, we could detect PRMT5 only in the BRG1 IP in proliferation condition. These data indicate that in proliferating myoblasts, BRG1 and BAF47 participate to the transcriptional repressive complex N-CoR-1, which is associated with KAP1 [48].

In early differentiating myoblasts, BRG1 is weakly detected with BAF47 while BAF53a is not detectable. Reciprocally, in the BRG1 IP, BAF47 is weakly detected while BAF53a appears as a clear band. These data suggest that BRG1 and BAF47 interactions are less abundant in differentiating myoblasts compared to proliferating myoblasts (Figure 5A). MyoD also co-immunoprecipitates with either BRG1 or BAF47 at this stage. We see the same amount of MyoD in both co-IPs in proliferating myoblasts, while in 24 h-differentiated cells we detect a lower amount in BAF47 IP than in BRG1 (while we precipitated the same amount of BRG1 and BAF47 in the two conditions) (Figure 5A). This observation could reflect modifications of complexes containing MyoD or changes in MyoD affinity for its partners. For both types of co-IP, using BRG1 or BAF47 antibodies, we could clearly detect KAP1 but not PRMT5 after 24 h of differentiation (Figure 5A).

In summary, our data suggest that: i) MyoD interacts with BRG1 and BAF47 in proliferating myoblasts and in early differentiating myoblasts; ii) BRG1 and BAF47 are involved in few types of complexes, at least SWI/SNF and N-CoR-1 repressive complex containing KAP1; iii) in early differentiating myoblasts, it seems that SWI/SNF complexes are less abundant and the involvement of BRG1 and BAF47 in N-CoR-1 repressive complexes is maintained.

To complete these studies we performed fractionation of soluble nuclear extracts on glycerol gradient followed by western blot analyses. We observed globally a similar distribution for BRG1 and BAF170 on one hand and KAP1 and HDACs on the other hand (Figure 5B). In proliferation conditions, BRG1 and BAF170 are mainly detected in fractions 6, 7 and 8 (Figure 5B). BAF47 is also detected in the same fractions even if it has a broader distribution (Figure 5B). KAP1 and HDACs are mainly detected in fractions 3, 4, 5 and 6 (Figure 5B). These fractions also contain MyoD and some levels of BAF47. Finally, BAF53a has a broad distribution pattern in proliferating and early differentiating myoblasts, being detectable in almost all fractions (Figure 5B).

The distinct distributions of BRG1 and BAF170 on one hand, KAP1 and HDACs on the other hand, most likely reflect their involvement in SWI/SNF and N-CoR-1 complexes. After 24 h of differentiation, distribution of BRG1 and BAF170 is enlarged, since the majority of these proteins are now detected in fractions 5 to 9 (Figure 5B). We cannot compare signal intensity between results between proliferating and early differentiating myoblasts since we cannot have correct standardization for fractionated extracts. KAP1 and HDAC proteins are mostly detected in fractions 4 to 7. BAF47 is clearly detected in all these fractions (4 to 9) and MyoD is mainly detected in fractions 4 to 7.

From all these results, we could point out an overlap in the distribution of BRG1 and BAF47 and the distribution of KAP1 and HDACs. This overlap most likely reflects the balance between SWI/SNF and N-CoR complexes that switch in favor of the N-CoR-1 complex upon differentiation.

## Discussion

The SWI/SNF complex remodels chromatin structure (via ATP-hydrolysis energy), once recruited to gene promoters by transcription factors or histone modifications, through disruption of DNA-histone interactions to activate/repress gene expression [7]. Skeletal muscle terminal differentiation is a two-step process starting with an irreversible cell cycle withdrawal followed by the activation of the muscle genetic program resulting in cell fusion and formation of multinucleated myotubes [36], [45], [49]. During terminal muscle differentiation, the muscle regulatory factors (MRFs) that belong to the bHLH family (MyoD, Myf5, MRF4 and myogenin) activate myogenic program by a well-orchestrated activation of muscle-specific genes [50], [51]. In this study, we sought to assess the role of two SWI/SNF BAF subunits (the tumor suppressor BAF47 and BAF53a) and the catalytic subunit BRG1 in skeletal muscle terminal differentiation. Indeed, previous works were mainly focusing on the role of the catalytic subunits (BRG1 and BRM) in differentiation programs, showing for example that BRG1 is actively involved in the expression of early [31] and late muscle genes [32]. BAFs are mainly considered as structural components of SWI/SNF complexes. However, recent papers showed that these subunits play important roles in a wide range of differentiation programs (11, 14, 29, 34, 35, 38). For example, BAF60c plays an important role in muscle differentiation in a two-step model. First, it mediates the initial recognition of the myogenin promoter by MyoD in myoblasts and, following its phosphorylation by p38α kinase, recruits the SWI/SNF complex [35].

In our study, we used siRNA-mediated downregulation of BRG1, BAF47 or BAF53a and analyzed the consequences on muscle differentiation and gene expression. Knockdown of BAF47, BAF53a or BRG1 impairs ex vivo myogenic differentiation of myoblasts. BAF53a-depleted cells exhibit almost standard levels of Myogenin, MCK and MHC since the observed decrease is not statistically significative. While in BAF47- or BRG1-depleted myoblasts, expression of muscle markers is largely decreased or even abolished. These results suggest different time-scale requirements for SWI/SNF subunits during differentiation. BAF53a being required in later steps for cell fusion but not so necessary for the appropriate program of early gene regulation, while BAF47 and BRG1 are required at early differentiation steps.

Several studies reported the role of HDACs in muscle differentiation [49], [52], [53], [54]. HDACs are frequently incorporated in larger protein complexes mainly, involved in transcription repression. Some of these repressor complexes, such as N-CoR and mSin3A/HDAC, also include SWI/SNF subunits [4] and other co-repressor factors. For example, it has been reported the presence of SWI/SNF members in the N-CoR-1 complex including BRG1, BAF170, BAF155, BAF47 and the co-repressor KAP1 [48], [55]. We have performed interaction experiments and we were able to co-precipitate BRG1, BAF47 and BAF53a from proliferating myoblasts, as well as HDAC 1–3 and KAP1, which are specific of the N-CoR-1 complex. Interestingly, we found a weaker association of BAF47 with SWI/SNF subunits in differentiating as compared to proliferating myoblasts, while KAP1/BAF47 interactions are more stable in differentiating and proliferating myoblasts. In any condition of IP, we also co-precipitate MyoD. Participation of BRG1 and BAF47 to SWI/SNF and N-CoR complexes was further assessed by complex fractionation on glycerol gradient. Comparing proliferating and differentiating myoblasts, we observed an increase of BAF47 in the fractions containing KAP1 and HDACs upon differentiation. Interactions between MyoD and SWI/SNF complexes [35] as well as with N-CoR factor [56] have been described, consistent with MyoD detection in nuclear extract fractions overlapping with BRG1 but also KAP1 distribution. Interaction of BRG1 and BAF47 with HDACs and KAP1 might be required for proper repression of transcription. Downregulation of these canonical subunits could affect both SWI/SNF and N-CoR-1 complexes functions, inducing a specific phenotype upon myogenic differentiation. We did not explore if BRM, the other ATPase subunit of SWI/SNF complexes could replace BRG1 in those complexes since these two enzymatic subunits are exclusive. It would be interesting if the BAF47 switch in the SWI/SNF and N-CoR-1 complexes could be correlated with involvement of BRM in the same complexes. From a personal communication from S. Albini it appears that BRM could play some specific roles during muscle terminal differentiation. Interestingly, N-CoR-1 has been implicated in muscle mass control and myogenesis [57]. Since BAF47 can participate to different type of complexes, either involved in chromatin remodeling like SWI/SNF or in transcription repression like N-CoR-1, it is likely that this subunit plays a dual and complex roles in the transcriptional control.

We were also interested in the role of individual SWI/SNF subunits in the irreversible cell cycle exit, since BRG1 and BAF47 are bona fide tumor suppressor genes. We have shown that these two subunits are present on *cyclin D1* promoter in proliferation and upon differentiation induction. Interestingly, BrdU incorporation experiments indicate that cell cycle exit upon serum deprivation is not total for BRG1 or BAF47-depleted myoblasts, while BAF53a-depleted cells behave as control cells. For BRG1, similar results were reported by de la Serna et al. [33]. Surprisingly, upon GM addition, only BAF47- but not BRG1-depleted cells were able to re-enter cell cycle. At a molecular level, cyclin D1 is re-expressed in BAF47-depleted cells. Re-expression of cyclin D1 in the absence of BAF47 is in agreement with a previous study that showed that BAF47 is targeted to cyclin D1 promoter along with HDAC1 to repress its expression [58]. The BAF47 effect on Cyclin D1 repression and cell cycle exit is specific and somehow not directly correlated to BAF47 requirement in myoblast differentiation since BRG1 and BAF53 do not modify permanent cell cycle exit but are, anyhow, essential in myoblast terminal differentiation. The different and complex roles played by SWI/SNF BAFs are far from being totally elucidated.The essential roles of BRG1 and BAF60c in myogenic differentiation have been already clearly demonstrated [31], [32], [35]. Our present study demonstrates that BAF47 and BAF53a are also required for proper myogenic differentiation. We have shown that BAF47 could play a dual role both in permanent cell cycle exit and muscle gene transcription and it participates in different complexes involved in transcription regulation. BAF47 participation to these complexes varies upon differentiation that could represent a fine mechanism to tune transcription. We have also highlighted a specific role for BAF47 compared to BRG1 in the control of the irreversible cell cycle withdrawal, even if both of them are known as tumor suppressor genes. The specificity of BAF47 in cell proliferation control is probably the reason why this tumor suppressor is inactivated in almost all the rhabdomyosarcomas, while the BRG1 inactivation is less frequent.

## Supporting Information

Figure S1 **siRNAs selection.** For each targeted SWI/SNF subunit, 4 siRNAs were tested for their downregulation efficiencies. Total protein extracts were prepared and analyzed by western blot. Samples were from proliferating C2C12 myoblasts 48 h post siRNA transfection. Scr = Scrambled control siRNA.
(EPS)

Figure S2 **Efficiencies of the downregulation assays described on** **Figure 3** **.** Total protein extracts were prepared from aliquots of cells used in the BrdU assay (Figure 4) and analyzed by western blot. Samples were from proliferating myoblasts (0) or induced to differentiate in DM for 96 h (96) or cells switched back to GM for 12 h (96+12 h GM).
(EPS)
